# Long-term Effect of Intra-Row Spacing on Growth and Productivity of Super-High Density Hedgerow Olive Orchards (cv. Arbequina)

**DOI:** 10.3389/fpls.2017.01790

**Published:** 2017-10-18

**Authors:** Maria Gomez-del-Campo, David J. Connor, Eduardo R. Trentacoste

**Affiliations:** ^1^CEIGRAM, Universidad Politécnica de Madrid, Madrid, Spain; ^2^Faculty of Veterinary and Agricultural Sciences, The University of Melbourne, Melbourne, VIC, Australia; ^3^Estación Experimental Agropecuaria Junín, Instituto Nacional de Tecnología Agropecuria (INTA), Mendoza, Argentina

**Keywords:** *Olea europaea* L., hedgerow orchard design, super-high density orchard, tree distance, hedgerow porosity

## Abstract

Intra-row spacing is known to determine early productivity of super-high density olive orchards depending on growing conditions, cultivar growth characteristics, planting geometry and subsequent pruning management but few experiments have been carried out in this olive hedgerow orchard design. In 2008 an experiment of 4-m spaced hedgerows was established with 8 intra-row spacings (from 1.0 to 2.5 m) in Toledo (Spain) resulting in orchards of density ranging from 2,500 to 1,000 trees ha^−1^. Tree growth was evaluated as height, trunk diameter and leaf area during the first 4 years. Hedgerow porosity was calculated from the 4th until the 9th year. In the 8th year hedgerow height, width, leaf area and branch angles were measured. Olives were harvested from 3rd to 9th year for measurements of fruit characteristics and productivity. Tree growth was not affected by intra-row spacing during the first 4 years. In the 8th year leaf area, external surface area and volume per tree were significantly greater in the more spaced trees; but hedgerow characteristics of leaf area per hectare, number of effective leaf layers horizontally through the hedgerow, and leaf density were not affected. In the more spaced trees insertion angles of branches to the vertical were significantly greater, mainly in the lower canopy. Intra-row spacing did not affect fruit characteristics. Oil production ha^−1^ decreased linearly with spacing during the first 4 harvests while production per tree increased significantly with spacing after the 3rd harvest. As a result, oil production ha^−1^ from the seven harvests combined only increased for tree spacing less than 1.2 m; wider spacing had no effect. Annual oil production ha^−1^ increased linearly as porosity was reduced by greater tree density and canopy development along the seasons.

## Introduction

The intensification of fruit orchards commenced in the 60s when semi-dwarfing and dwarfing rootstocks became available. The main objective of the transformation was to increase fruit production in the early years (Verheij, 1972; Webster, 2002). In olive orchards intensification started later and proceeded in two stages without the use of rootstocks. First, in the 70s, planting density was increased from 100 trees ha^−1^ in traditional orchards to 200–300 trees ha^−1^ in intensive ones but retained the same traditional vase-shaped canopies. Second, a much bigger change was added in the middle 90s with the introduction of super-high density orchards (1,500–2,500 trees ha^−1^) with low vigor cultivars grown to central leader in hedgerows to form continuous fruit-bearing canopies (Rallo et al., 2013; Connor et al., 2014).

Orchards must be designed at the outset for inter- and intra-row tree spacing, row orientation and cultivar(s) because there are severe limits to change once the orchard is established. Each of those decisions can contribute to determination of productivity and oil quality (Trentacoste et al., 2015a,b). In addition, tree density will affect profitability: first through the cost of planting and subsequent training of the trees in early years, and second through the time it takes the orchard to achieve maximum production. The first increases with density while the second decreases.

It has been observed in other fruit trees that plant density is the single most important factor affecting early yield, even more determinant than rootstock or training system (Robinson, 2003). Other effects of tree density should also be considered in order to determine the most suitable density. Tree growth is reduced in dense orchards because of root competition (Policarpo et al., 2006). Additionally, lower spacing between trees could reduce branching number and change the direction of shoot growth, as seen in forests (Valladares and Pearcy, 1998). In olive, crown architecture is responsive to illumination environment (Ventre-Lespiaucq et al., 2016), for example Rallo et al. (2013) observed more horizontally canopies in response to wide tree spacing.

Super-high density olive orchards managed as narrow hedgerows were developed in Spain in the 90s for harvest by modified grape-harvest machines. In the first orchards intra-row spacing was less than 1.2 m (Rius and Lacarte, 2015), although no experimental information was available to identify the most suitable intra-row spacing. The main objective of close intra-row spacing is to obtain rapid canopy development for early high fruit production, the timing of which also depends on cultivar vigor, climatic and soil conditions and orchard management (mainly irrigation and N fertilization). Cultivar vigor is also affected by fruit load since early fruiting reduces vegetative growth and hedgerow development (Castillo-Llanque and Rapoport, 2011; Fernández et al., 2015). Under high-vigor conditions (vigorous cultivar, optimum temperature and soil water content) trees can be planted at wider spacing than under low-vigor conditions and yet fill the hedgerow at the same time.

Genetic characteristics of the cultivar not only determine vigor but also growth habit and so the optimal training and intra-row spacing. The advantage of considering natural shape and tree architecture is to reduce manual training operations (Costes et al., 2006). Measurements of height and width that characterize the shape of olive trees place “Arbequina” in the “oval” group that are more adapted to successful training to the central axis of super-high density orchards than “round” cultivars (Moutier et al., 2008). Rosati et al. (2013) considered other growth aspects that determine adaptation to high-density orchards. These authors compared tree architecture of 21 olive cultivars and observed that cultivars, such as Arbequina and Arbosana, characterized by thin branches inserted at high angles, developed higher yield efficiency, i.e., flower number trunk diameter^−1^. In another experiment Camposeo and Godini (2010) observed that the compact shape of some cultivars, as in cv. Urano, was well adapted to super-high density orchards.

The long-term data required for reaching conclusions on orchard design can explain why few experiments have been carried out in olive or other fruit trees to determine optimum intra-row density, compared with annual crops (e.g., Shapiro and Wortmann, 2006). In olive most density trials have compared combinations of inter- and intra-row spacing (Rallo et al., 2013). No experiment has concentrated on tree and orchard yield response to intra-row spacing.

In 2008 an experiment to evaluate the effect of intra-row distance on olive hedgerow growth and production was established in Toledo (Spain). Vegetative growth (height, width, leaf area, trunk diameter and stem angles) and yield components (fruit mass, oil content and fruit number) of individual trees and entire hedgerow were evaluated over 9 years from planting.

## Materials and methods

### Site and orchard

The experimental olive (cv. Arbequina) hedgerow orchards were planted N-S in June 2008 near La Puebla de Montalbán (39° 53 N, 4° 27W, 479 masl), Toledo, central Spain. The hedgerow experiment consisted of 3 rows with 72 trees established at 4 m inter-row space. There were three replicates of 8 intra-row spacing, 1.0, 1.125, 1.375, 1.625, 1.875, 2.125, 2.375, 2.50 m that correspond to orchard densities of 2,500, 2,222, 1,818, 1,538, 1,333, 1,176, 1,053, 1,000 trees ha^−1^, respectively. Data were collected from the central rows.

The soil was a clay-loam of three layers; an A horizon (0–0.15 m), a Bt horizon (0.15–0.40 m) and below a carbonaceous C horizon which impeded root penetration. The hedgerows were managed using standard commercial practice. Supplementary irrigation was applied from March to October using single drip lines per row. In 2008, at planting, one emitter discharging 3.0 L h^−1^ was located at each tree. In 2010 a second emitter was added at each tree. Then from 2012 the drip line was replaced with on with emitters (3.0 L h^−1^) at 0.5 m spacing was installed along all rows. Irrigation was applied to ensure that trees did not suffer water stress, for this both reference evapotranspiration and rainfall were considered. Fertigation was used to apply nutrient amounts determined by prior leaf analysis. Mean annual applications were 150 kg ha^−1^ of N and K and 90 kg ha^−1^ of P. From experimental planting, a central leader was trained on bamboo stake of 2.5 m height until 2012. From planting (spring 2008) until winter 2011-2012, shoots developed on each trunk were pruned and the terminal apex was trained in order to achieve rapid continuous canopy walls. From the end of season 2012 (winter 2012-2013) branches extending into the alleys were removed annually to facilitate the passage of the harvester. After 2013, hedgerows were mechanically topped annually to 2.5 m height.

A weather station at the site registered wind speed and direction, rainfall, temperature, humidity and global radiation at 30-min intervals and calculated reference evapotranspiration (ETo) by the Penman–Monteith method (Allen et al., 1998).

### Hedgerow vegetative structure

Initial increase of tree height and trunk diameter was measured from 1-year after planting (2008) to 3-years after planting (2011): 24/6/2008, 24/4/2009, 4/3/2010, 7/10/2010, 9/5/2011, 25/11/2011 on three trees per treatment to establish when tree crowns achieved the target height (i.e., 2.5 m) optimal for the harvesting machine. Tree leaf area was estimated by measuring the diameter of all branches in 3 trees per treatment and applying the regression:

(1) $$\begin{array}{r} \text{Leaf area }\left({\text{m}}^{2}\right)=0.00099408\;x^{2.1772};R^{2}=0.97^{**} \end{array}$$

where *x* is branch basal diameter, less than 55 mm. The leaf area equation was obtained from 47 branches of trees on the experimental site. For this, basal diameter of each branch was measured and all leaves were removed and area determined with a leaf area meter (Li-3100; Li-Cor, Lincoln, NE, USA).

The foliage architecture of three trees per intra-row spacing treatment was described in detail after 2015 harvest (8th year after planting). Height of top and bottom foliage was measured in 3 positions per tree near the trunk and at 0.5 m on each side. Hedgerow width was measured at 0.5, 1.0, and 1.5 m height at three positions on the same trees. Hedgerow external surface area and canopy volume were calculated for rectangular shapes. Hedgerow leaf area was estimated by measuring the diameter of all branches on all studied trees and applying the Equation (1). From this, leaf area density was calculated as tree leaf area canopy volume^−1^, and the number of effective leaf layers horizontally through the hedgerow was estimated as tree leaf area hedgerow surface^−1^. Insertion angle from the vertical was measured for branches inserted at different heights on the axis (0.8, 1.5, and 2 m).

**Figure 1:**
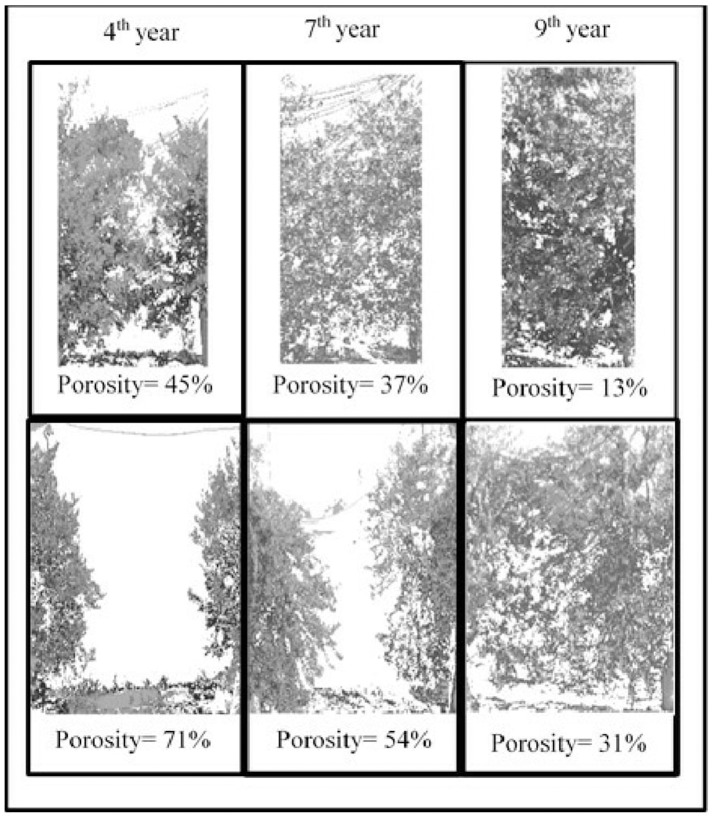
Digital image analysis for estimation of hedgerow porosity. All hedgerows are spaced at 4 m. Upper and lower panels are hedgerows with intra-row spacing of 1.13 m (upper panels) and 2.38 m (down panels) in the 4th, 7th, and 9th year after planting, respectively. The images were processed to discriminate between canopy cover and gaps between trees. Porosity (%) was determined from the ratio of the number of red pixels (background sheet) to the total number of pixels in the image.

Hedgerow porosity was measured in two trees per intra-row spacing treatment in winter 2011, 2012, 2014, 2015, and 2016 by image analysis of digital pictures. The camera was positioned perpendicular to the hedgerow surface and photos from trunk to trunk of contiguous trees were taken against the background of a red sheet placed on the opposing face of the hedgerow. Photographs were processed digitally using CobCal software ver. 2.0 (Bs As, Argentina) to estimate average percentage of gaps by dividing the number of red pixels (i.e., background sheet) by the total number of red and green (leaves and stems) pixels in the image (Figure 1). The number of seasons needed for overlapping of shoots from contiguous trees was determined visually from photos. Because we only had two images of good quality per treatment and year, porosity data were not subjected to analysis of variance.

### Oil yield and its components

Olives were harvested from three individual trees per intra-row spacing on 19/10/2010, 28/10/2011, 17/10/2012, 7/11/2013, 19/10/2015, and 2/11/2016. Due to climatic conditions there was no production in 2014. Subsamples of 25 g were weighed fresh and maturity index (MI) was determined from color of skin and pulp (Uceda and Frías, 1975). In 2010 and 2011, three subsamples per tree of 25 g were dried in a forced-air oven at 105°C for 42 h for determination of fruit dry weight. In 2012 and 2013 and in 2015 and 2016, 6 and 15 subsamples, respectively, of 25 g were measured. Fruit oil content was measured in dry subsamples by nuclear magnetic resonance (MiniSpec MQ-10; Bruker, Madison, WI, USA) using the method described by del Río and Romero (1999). The number of fruits per tree was estimated from total fresh fruit yield and average fruit fresh weight. Oil production was calculated as the product of fruit number and fruit oil content. Orchard productivity (per ha) was calculated according to tree density.

The effects of intra-row spacing on measured parameters were compared using a randomized block complete design with three replicates. The means were separated using the LSD-test for a level of significance *P* ≤ 0.05. Regression analysis was used to determine association among parameters.

## Results

### Environmental conditions

**Figure 2:**
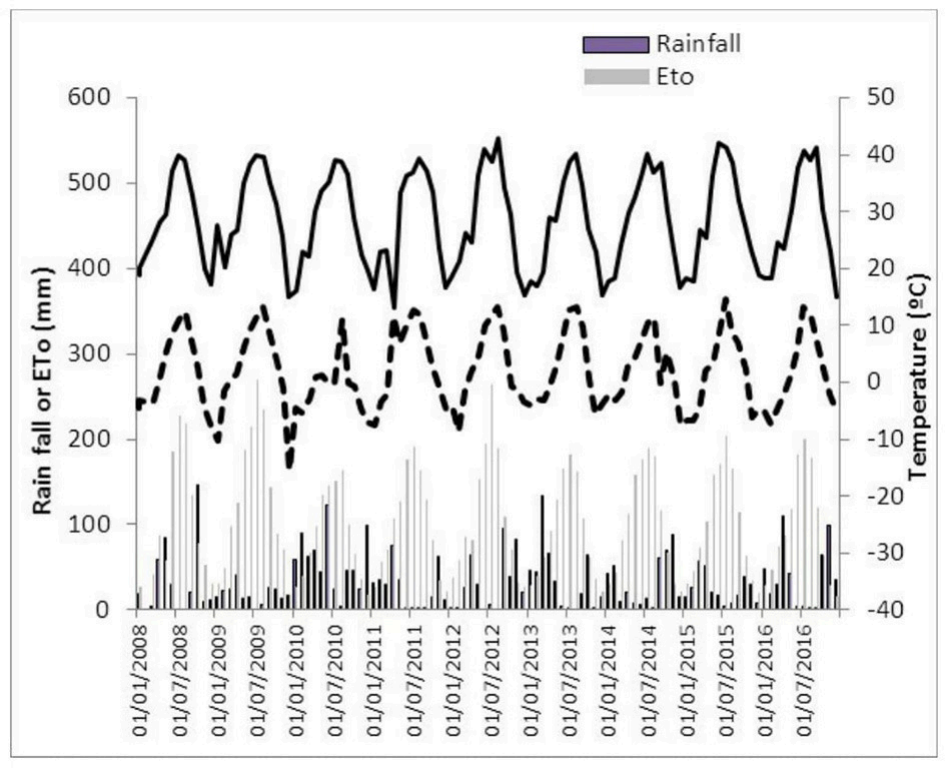
Monthly rainfall, reference evapotranspiration, and minimum and maximum temperatures from January 2008 until December 2016 at the experimental orchard located in Puebla de Montalbán (Toledo, Spain).

Weather data are summarized in Figure 2 as mean monthly minimum and maximum temperatures (°C) and monthly totals of rainfall (mm) and reference evapotranspiration (ETo, mm). During the experimental years mean temperature, rainfall and ETo were 15.2°C, 395 and 1205 mm, respectively. July and August were the hottest months and December and January the coldest. Highest temperature (43°C) was recorded in August 2012 and lowest (−15°C) in December 2009. Rainfall varied between years, from 212 mm in 2009 to 693 mm in 2010.

### Vegetative growth

**Figure 3:**
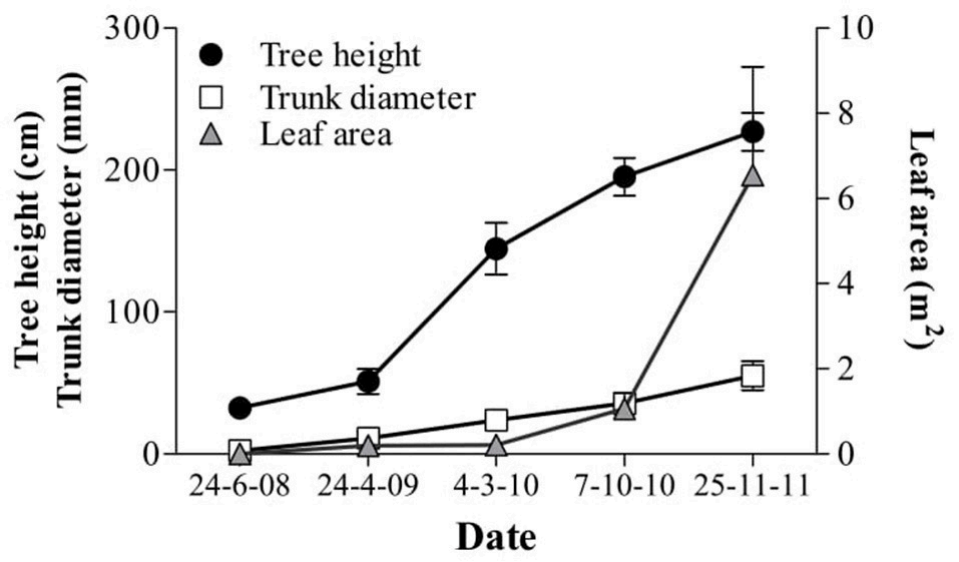
Average components of vegetative growth of trees established at eight different intra-row spacing during 4 years after planting. Each point is a mean of 3 replicates for 8 intra-row spacings (*n* = 24) ± standard deviation. No significant differences were observed among spacing treatments.

Intra-row spacing did not affect any vegetative growth parameter of individual trees during the first 4 years (Figure 3) with mean coefficients of variability of 10, 20, and 40% for height, trunk diameter and leaf area, respectively. The vegetative parameters presented different patterns during the early years. Trees gained most height during the 2nd growing season but most leaf area during the 4th season, while trunk diameter increased progressively from the 2nd until the 4th season. During the following 4 years (5th–8th year) height, trunk diameter and leaf area increased slowly by 0.3 m, 22 mm and 3.3 m^2^, respectively (Tables 1, 2). In the 8th year no significant differences were observed in hedgerow height, width or trunk diameter. Meanwhile tree leaf area, hedgerow external surface area and volume at tree individual level were greater in the more widely spaced treatments. However, hedgerow external surface area or volume per ha level was significantly greater at smaller spacing (from 1.0 to 1.63 m intra row-spacing) in which hedgerows were completely covered. Leaf area per ha was not significantly affected. Leaf layers and leaf density were not significantly affected. Intra-row spacing significantly modified stem growth habit. In all experimental trees the greatest angle to vertical was observed in the lower branches at 0.8 m of height (155°) compared to 72° at 2 m of height. In the more widely spaced trees the mean angle to the vertical (129°) was greater compared to smallest spacing (90°), due mainly to differences in the bottom layer of the hedgerows.

**Table 1:** Characteristics of hedgerow trees in the 8th year after planting at various intra-row spacing.

**Intra-row spacing**	**Hedge height**	**Hedge width**	**External surface area**		**Canopy volume**		**Leaf area**		**Leaf layers**	**Leaf density**
**(m)**	**(m)**	**(m)**	**(m^2^ tree^−1^)**	**(m^2^ ha^−1^)**	**(m^3^ tree^−1^)**	**(m^3^ ha^−1^)**	**(m^2^ tree^−1^)**	**(m^2^ ha^−1^)**	**(number)**	**(m^2^ m^−3^)**
1	2.28	1.23	5.78d	14,444a	2.80d	6,998abc	4.31c	10,771	1.53	1.22
1.125	2.23	1.53	6.17cd	13,708a	3.80cd	8,449a	12.01ab	26,690	3.55	2.27
1.375	2.28	1.36	6.42bc	11,666b	4.23bc	7,689abc	6.68bc	12,145	2.19	1.50
1.625	2.26	1.44	6.81ab	10,475c	5.17ab	7,949ab	12.55ab	19,305	2.86	1.97
1.875	2.31	1.48	7.07a	9,433d	5.68a	7,575abc	8.22abc	10,965	1.74	1.19
2.125	2.23	1.40	6.80ab	8,000e	5.22ab	6,144bcd	9.33abc	10,979	1.99	1.48
2.375	2.25	1.53	7.05a	7,423ef	5.74a	6,042cd	11.44ab	12,044	2.59	1.69
2.5	2.29	1.42	6.77abc	6,769f	5.04ab	5,047d	13.77a	13,766	3.09	2.19

*Each value is a mean of 3 replicates. Values with the same letter are not significantly different among intra-row spacing by LSD test, P ≤ 0.05. Letters only presented when ANOVA indicated significant effect*.

**Table 2:** Structure of trunk and branches in the 8th year of trees planted at various intra-row spacing.

**Intra-row spacing (m)**	**Trunk diameter (mm)**	**Stem angle at 0.8 m high (°)**	**Stem angle at 1.5 m high (°)**	**Stem angle at 2.0 m high (°)**	**Mean stem angle (°)**
1.00	69	136ab	82	54	90c
1.13	70	121b	90	71	94c
1.38	75	118b	97	48	88bc
1.63	85	138ab	107	71	105abc
1.88	81	170a	111	88	123a
2.13	75	150ab	106	93	116ab
2.38	77	180a	97	86	121a
2.50	82	175a	125	86	129a

*Each value is a mean of 3 replicates. Values with the same letter are not significantly different among intra-row spacing by LSD test, P ≤ 0.05. Letters only presented when ANOVA indicated significant effect*.

**Figure 4:**
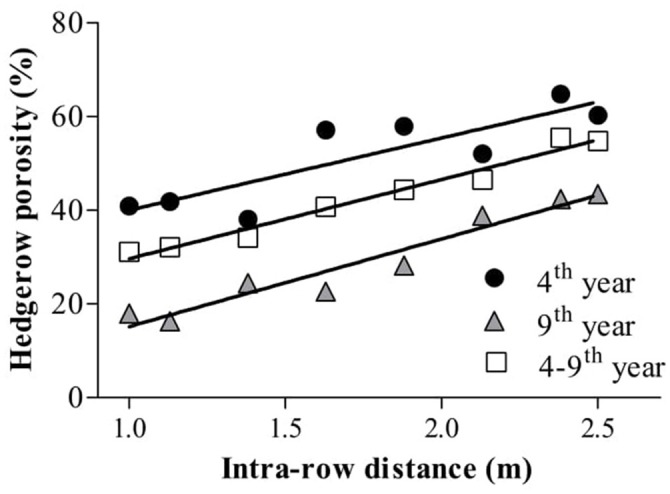
Hedgerow porosity in the fourth and ninth year after planting trees at various intra-row distances. Average porosity from the fourth to the ninth year is also shown. Each point is a mean of 2 replicates for 8 intra-row spacings (*n* = 16). The parameters of the model for 4th year were: y = 24.6+15.4, *R*^2^ = 0.74; for 9th year: y = −3.5+18.7x, *R*^2^ = 0.94; and for 4–9th: y = 12.8+16.9x, *R*^2^ = 0.98, where y is hedgerow porosity.

Evolution of hedgerow porosity responded to intra-row spacing and season (Figures 1, 4), varying over the experimental period from 60 to 10% (Figure 4). The greatest values were observed in young trees at wide spacing and the smallest in closely spaced old trees. Linear relationships were established between hedgerow porosity and tree intra-row spacing (*R*^2^ = 0.74, 0.94, and 0.97 for 4- and 9-year-old trees and mean 4–9th years). Porosity decreased from 2011 to 2016 but the response to intra-row spacing remained relatively constant. Intra-row spacing of 1.0 and 1.13 m required three growing season for contiguous plants to overlap. For wider spacings, the durations were four, six, seven and nine seasons, for 1.38, 1.63, 1.88–2.38, and 2.5 m, respectively.

### Production and fruit characteristics

First harvest was made at the end of the 3rd growing season. During the next 7 years, except for 2014 (7th year), fruit characteristics and production were evaluated (Tables 3, 4). Production per tree increased at each spacing treatment until the 4th year, after which increase was restricted to the most widely spaced trees (2.5 m).

**Table 3:** Fruit characteristics for the first 6 years of harvest of trees planted at various intra-row spacing.

**Intra-row spacing (m)**	**Fruit fresh weight (g)**	**Fruit dry weight (g)**	**Oil content (% fw)**	**Oil content (% dw)**	**Oil content (g fruit^−1^)**	**MI**
**2010 (3RD YEAR)**						
1.00	2.03	0.83	17.5	42.7	0.36	1.88
1.13	2.00	0.83	17.2	41.6	0.34	1.16
1.38	2.44	0.98	17.4	43.6	0.43	3.22
1.63	1.96	0.82	18.1	43.1	0.35	1.72
1.88	2.03	0.83	17.5	42.7	0.36	1.88
2.13	1.97	0.83	18.1	42.9	0.36	1.52
2.38	2.06	0.85	17.5	42.5	0.36	1.82
2.50	1.98	0.81	17.3	42.3	0.34	1.53
**2011 (4TH)**						
1.00	1.31	0.60	20.2	43.7	0.26	0.86
1.13	1.77	0.84	22.7	48.0	0.41	2.34
1.38	1.24	0.55	18.2	40.9	0.23	1.38
1.63	1.57	0.73	21.6	46.7	0.35	1.99
1.88	1.78	0.83	22.6	48.9	0.40	1.65
2.13	1.85	0.87	21.9	46.9	0.41	1.83
2.38	1.38	0.60	19.5	44.8	0.27	1.10
2.50	1.54	0.66	20.0	46.7	0.31	1.25
**2012 (5TH)**						
1.00	1.50bc	0.64bc	19.1	45.0	0.29bc	0.44ab
1.13	1.39c	0.58c	18.6	44.7	0.26c	0.29c
1.38	1.90a	0.79a	19.5	46.7	0.37a	0.43abc
1.63	1.61b	0.69b	20.0	46.7	0.32b	0.51a
1.88	1.50bcd	0.62bc	19.2	45.0	0.28bc	0.46a
2.13	1.46cd	0.61c	19.0	45.2	0.28c	0.50a
2.38	1.54bc	0.63bc	19.0	46.1	0.29bc	0.33bc
2.50	1.43d	0.59c	18.4	44.6	0.26c	0.29abc
**2013 (6TH)**						
1.00	1.12	0.46	17.5	42.8	0.20	0.29
1.13	1.12	0.45	16.7	41.1	0.19	0.29
1.38	1.13	0.45	16.8	42.3	0.19	0.31
1.63	1.15	0.46	16.7	41.7	0.19	0.34
1.88	1.22	0.49	17.5	43.3	0.21	0.32
2.13	1.15	0.46	16.9	41.9	0.19	0.25
2.38	1.14	0.46	17.2	42.9	0.20	0.44
2.50	1.09	0.44	17.0	41.7	0.19	0.36
**2015 (8TH)**						0.10
1.00	1.34	0.55	15.6	37.7	0.21	0.08
1.13	1.20	0.49	14.9	36.3	0.18	0.07
1.38	1.24	0.51	15.0	36.7	0.19	0.09
1.63	1.32	0.54	15.1	36.5	0.20	0.08
1.88	1.29	0.52	15.2	37.5	0.20	0.10
2.13	1.23	0.51	15.7	38.1	0.19	0.10
2.38	1.25	0.51	15.5	37.9	0.19	0.08
2.50	1.22	0.51	16.1	38.5	0.19	0.10
**2016 (9TH)**						
1.00	1.39	0.52	15.8	42.2	0.22	0.16
1.13	1.36	0.50	15.9	43.1	0.22	0.17
1.38	1.40	0.52	15.9	42.7	0.22	0.15
1.63	1.44	0.52	15.4	42.4	0.22	0.15
1.88	1.49	0.57	17.2	44.7	0.26	0.15
2.13	1.58	0.59	17.1	45.5	0.27	0.15
2.38	1.42	0.57	18.2	45.3	0.26	0.15
2.50	1.39	0.57	18.4	45.4	0.26	0.16

*Each value is a mean of 3 replicates. Values with the same letter are not significantly different among intra-row spacing within each year by LSD test, P ≤ 0.05. Letters only presented when ANOVA indicated significant effect*.

**Table 4:** Fruit and oil production for the first 6 harvests of trees planted at various intra-row spacing.

**Intra-row spacing**	**Fruits**	**Fruit production**		**Oil production**	
**(m)**	**(# tree^−1^)**	**(g tree^−1^)**	**(kg ha^−1^)**	**(g tree^−1^)**	**(kg ha^−1^)**
**2010 (3RD YEAR)**					
1.00	3	6	15	1	3
1.13	69	138	306	24	53
1.38	208	508	924	89	161
1.63	658	1,246	1,917	232	357
1.88	0	0	0	0	0
2.13	553	1,088	1,279	197	232
2.38	340	700	737	122	129
2.50	77	155	155	27	27
**2011 (4TH)**					
1.00	3,238	4,180	10,450a	844	2,111a
1.13	1,814	2,752	6,115ab	594	1,320ab
1.38	4,082	5,039	9,163ab	925	1,682ab
1.63	3,171	4,821	7,417ab	1,031	1,586ab
1.88	2,314	4,043	5,390ab	914	1,219ab
2.13	2,087	3,377	3,973b	730	859b
2.38	4,196	5,776	6,080ab	1,133	1,192ab
2.50	3,302	4,892	4,892ab	965	966ab
**2012 (5TH)**					
1.00	2,355bc	3,574bc	8,934	686cd	1,716
1.13	2,786bc	3,831bc	8,514	717cd	1,594
1.38	1,277c	2,420c	4,400	473d	860
1.63	2,945bc	4,751bc	7,310	949bc	1,460
1.88	3,627ab	5,254ab	7,005	1,009bc	1,345
2.13	4,588a	6,571a	7,731	1,235ab	1,453
2.38	4,844a	7,470a	7,864	1,414a	1,488
2.50	4,205ab	5,967ab	5,967	1,099abc	1,099
**2013 (6TH)**					
1.00	6,323d	7,050cd	17,625a	1,227d	3,067a
1.13	5,968d	6,655d	14,789ab	1,112d	2,472ab
1.38	6,790cd	7,669bcd	13,944ab	1,294cd	2,353b
1.63	7,517cd	8,568bcd	13,182b	1,431bcd	2,202b
1.88	8,441bcd	10,315abc	13,753ab	1,806abc	2,409ab
2.13	9,720abc	11,168ab	13,139b	1,883ab	2,215b
2.38	11,470ab	13,057a	13,744ab	2,239a	2,357b
2.50	11,642a	12,790a	12,790b	2,171a	2,171b
**2015 (8TH)**					
1.00	4,029c	5,380d	13,451	839c	2,097
1.13	5,841bc	6,810cd	15,133	1,002c	2,227
1.38	5,092c	6,327cd	11,503	951c	1,729
1.63	6,169bc	7,936bcd	12,209	1,193bc	1,835
1.88	7,069bc	8,990bc	11,986	1,395bc	1,860
2.13	7,068bc	8,624bcd	10,146	1,345bc	1,582
2.38	9,275ab	11,451ab	12,054	1,787ab	1,882
2.50	11,076a	13,075a	13,075	2,103a	2,103
**2016 (9TH)**					
1.00	5,447c	7,529c	18,823	1,192c	2,981
1.13	5,001c	6,676c	14,837	1,032c	2,293
1.38	4,838c	6,813c	12,388	1,107c	2,013
1.63	4,705c	6,810c	10,478	1,090c	1,677
1.88	7,329abc	10,599abc	14,132	1,765bc	2,353
2.13	6,387bc	9,868bc	11,609	1,674c	1,969
2.38	10,399a	14,522a	15,286	2,617a	2,755
2.50	9,778ab	13,560ab	13,560	2,504ab	2,504

*Each value is a mean of 3 replicates. Values with the same letter are not significantly different among intra-row spacing within each year by LSD test, P ≤ 0.05. Letters only presented when ANOVA indicated significant effect*.

Fruit characteristics (fruit weight, oil content and MI) were not significantly affected by intra-row spacing in any season except in the fifth year (Table 3). Over all harvests, average fruits were 1.50 and 0.62 g fresh and dry weight; oil content was 0.27 g of oil per fruit (43 and 18% per dry and fresh weight, respectively) and, most of fruits were colored green (MI = 0.71).

**Figure 5:**
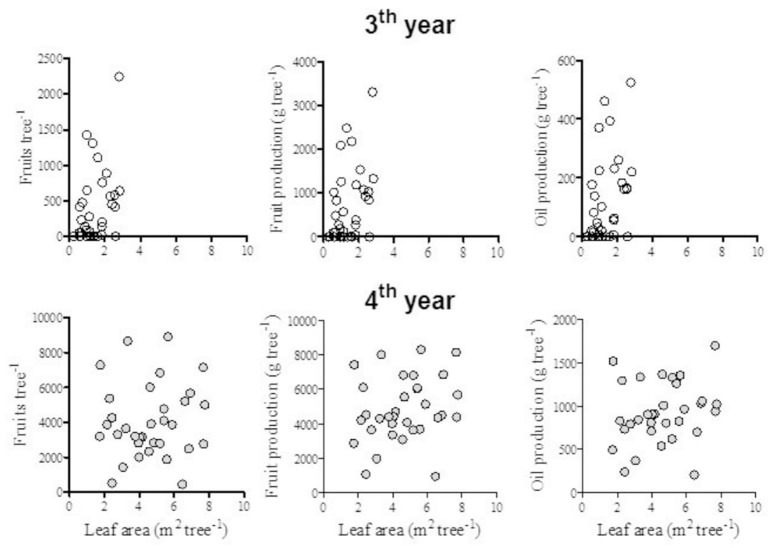
Relationships between fruit number and leaf area during the 3rd and 4th years of production in the experimental trees at various intra-row spacing. Points correspond to individual trees.

Fruit mass and number per tree were not significantly affected by intra-row spacing during the first 2 years (Tables 3, 4). After the 5th year, mass and number of fruits of the most widely spaced trees (2.5 m) were nearly double that the most closely spaced trees (1 m). Meanwhile fruit and oil production per ha were significantly greater in the least spaced treatment in the 4th and 6th year. During the 3rd and 4th years tree production was highly variable (coefficient of variability of 135 and 36%, respectively). The relationship of the first 2 years production and leaf area was evaluated. There was no significant relationship of fruit mass, fruit number, or oil production with tree leaf area (Figure 5), nor with trunk diameter (data not shown).

**Figure 6:**
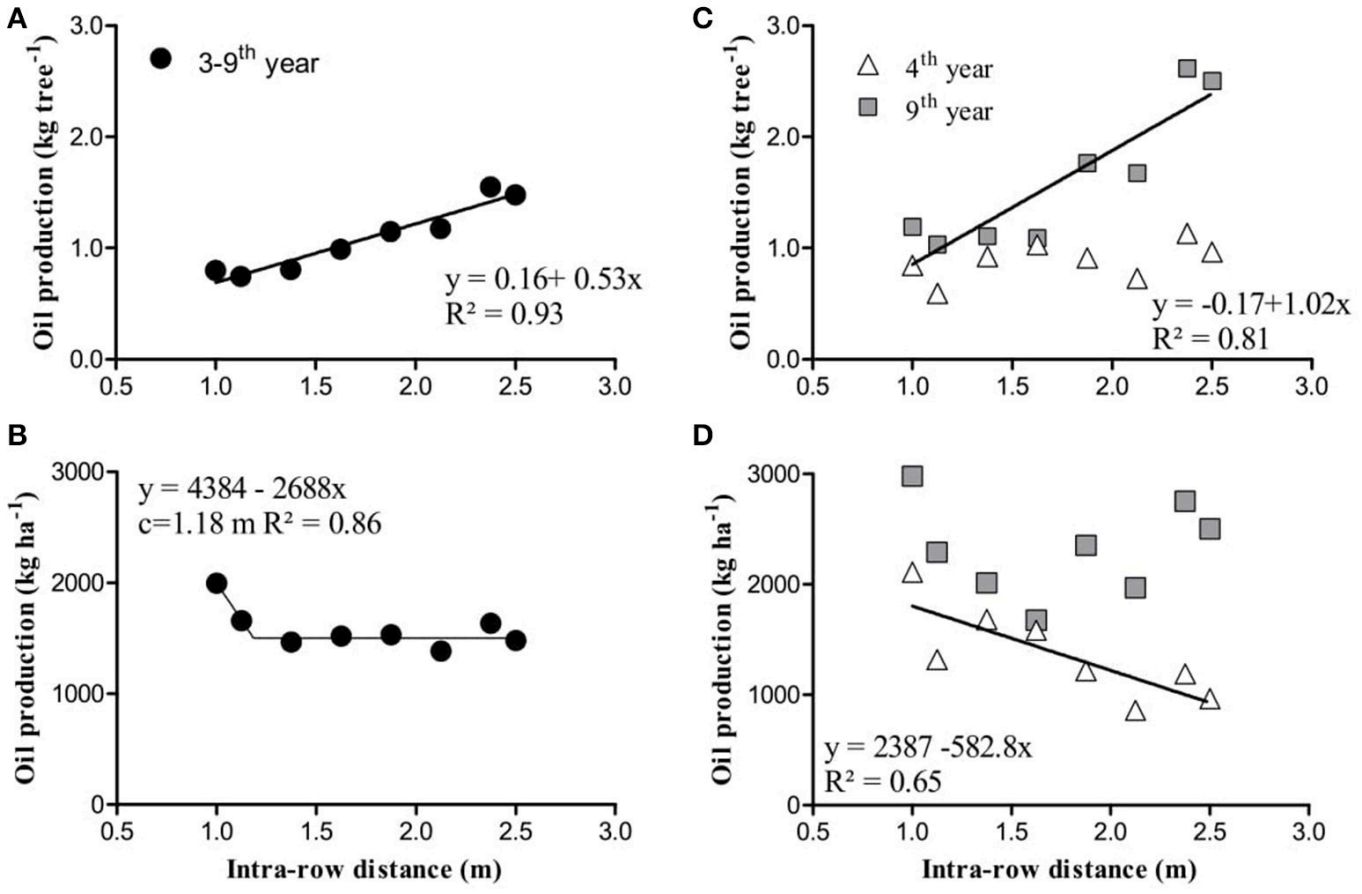
Mean oil production of olive hedgerows established with trees at various intra-row distances; per tree **(A)** and per ha **(B)** for the first seven harvested years, and in the fourth and ninth year after planting per tree **(C)** and per ha **(D)**. Each point is a mean of 3 replicates.

Oil production, per tree or ha, presented different relationships with intra-row spacing that also differed between years (Figure 6). Mean oil production per tree increased linearly from 3rd to 6th and from 3rd to 9th year with intra-row spacing (y = 0.27 + 0.36x; *R*^2^ = 0.88; y = 0.16 + 0.53x; *R*^2^ = 0.93, respectively Figure 6A). In contrast, from 3rd to 6th year (four harvests) oil production ha^−1^ decreased linearly with intra-row spacing increase (y = 1778 – 263x; *R*^2^ = 0.58). Similarly, a linear relationship was obtained between mean production during the first 4 harvests (kg ha^−1^) and tree density (trees ha^−1^) (y = 867 + 0.29x; *R*^2^ = 0.67). This relationship informs that yield increases by 1.14 kg oil ha^−1^ for each additional tree during the first 4 years. Oil production per ha decreased from 3th to 9th years by 538 kg as intra-row spacing was reduced from 1.0 to 1.2 m. At greater tree spacing there was no effect on oil production (Figure 6B).

But these relationships varied between individual years. During the 4th year, production per tree was not related with intra-row spacing (Figure 6C) but a negative relationship was observed with production per ha (y = 2387 − 583x; *R*^2^ = 0.64, Figure 6D). In contrast, in the 9th year production per tree increased with tree spacing (y = − 0.17 + 1.02x; *R*^2^ = 0.81, Figure 6C) but there was no relationship between production per ha and tree spacing (Figure 6D).

**Figure 7:**
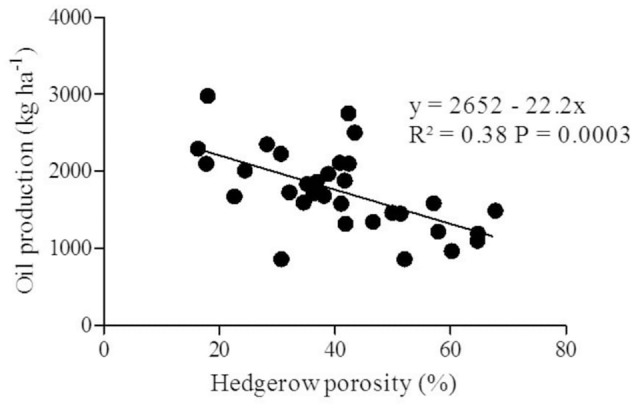
Relationship between oil production (kg ha^−1^) and hedgerow porosity (%) from the fourth until ninth after planting trees established at different intra-row spacing. Each point corresponds to two replicate trees per intra-row treatment in the various study years.

Hedgerow porosity was reduced throughout the experimental period by canopy development and intra-row spacing (Figure 1). Extreme values (Figure 7) were 68% (4th year at 2.38 m spacing) and 16% (9th year at 1.13 m spacing). Considering all years and intra-row treatments, the results reveal that oil production per ha was negative and linearly related with hedgerow porosity (*R*^2^ = 0.38, Figure 7), such that reduction in porosity by 10% was associated with increased oil production of nearly 220 kg ha^−1^.

## Discussion

Tree density can be increased by reducing distance between rows (inter-row spacing) and/or between trees within rows (intra-row spacing). Closer rows, that should allow radiation to reach the lowest part of the hedgerow, can be achieved with a ratio between canopy depth to free alley width close to unity (Trentacoste et al., 2015a). To the present time, the response to intra-row in olive orchards has only been studied in combination with different inter-row distances (León et al., 2007; Larbi et al., 2012; Díez et al., 2016).

In this study the effect of intra-row spacing was evaluated during 9 years in an experimental orchard established in 2008 in Toledo, Spain. The site is characterized by low rainfall (395 mm), high ETo (1,205 mm) and low minimum temperature in autumn and winter (e.g., −15°C in December 2009) (Figure 2). Irrigation and orchard management, however, allowed trees to form hedgerows topped to 2.5 m in the third season with high oil production (>2,000 kg oil ha^−1^) except in 2014 when flowering was seriously reduced by the dry summer and autumn conditions in the previous year. Trees were trained to a central leader that forced them to grow quickly in height during the 2nd and 3rd years, while the largest development of leaf area occurred during the 4th year (Figure 3). This growth is in accordance with experience at other experimental sites where trees also formed canopies of 2.5–3.00 m height in 3 years (Connor et al., 2014). Here, irrigation management focused on avoiding water limitations in all intra-row spacing treatments. Future studies could usefully explore the effect of intra-row spacing on plant water relations seeking a more efficient water use. For this, the irradiance model developed by Connor et al. (2016) could be a practical tool.

In the first 4 years, vegetative growth of individual trees, evaluated as height or leaf area (Figure 3), was not significantly affected by intra-row spacing whereas by the 8th year leaf area, external surface area and canopy volume per tree were significantly reduced with increasing density (Tables 1, 2). This suggests that competition between trees started after the 4th year when olives had achieved the maximum height.

Trunk diameter is a convenient measurement that has been well correlated with tree volume (Moutier et al., 2008). In this experiment, however, it was not significantly affected by tree spacing (Table 2) even in the 8th year (Figure 2 and Table 2) when large differences in tree volume and also leaf area were observed. This lack of response of trunk diameter to competition between trees could be related with (i) small trunk diameter of cv. Arbequina compared with other olive cultivars (Rosati et al., 2013; Marino et al., 2017); (ii) the experimental conditions characterized by a short growing season, shallow soils and super-high density (1,000–2,500 trees ha^−1^) and (iii) distances between trees being less than in other experiments in which a wider density range (312–1,250 trees ha^−1^), also in cv. Arbequina, was shown to reduce trunk cross section from 5th year after planting (Larbi et al., 2012).

Arbequina is well adapted to training with a central leader for hedgerows because its oval growth (Marino et al., 2017) developed with more, and thinner, lateral branches and shoots with more nodes than other cultivars (Rosati et al., 2013). These characteristics allow the axis to be well covered with many leaves and buds. In this experiment under super-high density management we observed that branch insertion angle differed along the axis, being greatest in the lower part and least in the higher part of canopy (Table 2), consistent with observations reported by Camposeo and Godini (2010). However, intra-row spacing was also shown to modify the insertion angle of branches. Here, branches of trees under wider spacing grew more horizontally than those in more narrow spaced trees, particularly in the lower part of the canopy. This modification allows more widely spaced trees to develop a round-shaped canopy with significantly larger external surface area (Table 1).

When hedgerow characteristics were evaluated in the 8th year no significant differences were observed in leaf area per ha. But external surface area and canopy volume ha^−1^ were greater in more dense treatments (Table 1). Canopy development described by hedgerow porosity could explain this difference (Figure 4). Porosity decreased as canopies developed, always with the smallest values at the closest tree spacing.

Porosity is a relatively easy measure of hedgerow structure that shows how canopies of hedgerows of varying tree densities develop in time as trees grow (Figure 4). Here, porosity ranged from 60% in the 4th year for the 2.5-m spaced trees to 18% in the 9th year for 1-m spaced trees. Porosity integrates differences in leaf angles, leaf shape and leaf area density (Mariscal et al., 2000; Castillo-Ruiz et al., 2016) making it a valuable canopy descriptor radiation interception (Oyarzun et al., 2007; Connor et al., 2009) that is shown here to be linearly related to oil production when all data were pooled (Figure 7).

The results reveal that it takes 4 years to achieve a continuous hedgerow for trees spaced less than 1.5 m and 9 years for trees spaced at 2.5 m (Figure 4). These periods could be termed “lost time” in the sense of productivity. Agronomic practices that increase capture of available resources such as radiation, water and nutrient can shorten this “lost time” with consequent greater production and efficiency of resource use (Steiner, 1986). In this experiment, the more spaced trees required many years to achieve continuous hedgerows suitable for over-row harvesters because strong branches entering the intra-row space were removed.

Olives are characterized by low production during the early years, compromising the profitability of the crop. Genetic characteristics and tree density are the main factors that can minimize this negative aspect. Cultivar Arbequina is considered an early producing cultivar (Marino et al., 2017), even though the first harvest here was in the 3rd season. Production in that year was small (mean 297 kg oil ha^−1^) but increased to more than 850 kg oil ha^−1^ in the 4th year (Table 4). The high variability of production during the first seasons (coefficients of variation of 135 and 36% in the 3rd and 4th years, respectively) should be considered in future experiments with young trees in order to increase the number of repetitions. The relationship between yield components and leaf area was studied in an attempt to understand this high variability in yield, but no relationship could be established (Figure 5). This suggests that those management techniques that induce vigor will not ensure high production in the first years and points to physiological responses apart from vegetative growth that also determine production of young trees.

During the first 4 years production per tree was not significantly affected by intra-row spacing (Table 4). After the 5th year, however, a positive linear relationship was observed with spacing (Figure 6A) such that number of fruits and oil production per tree was significantly greater in the more widely spaced trees. León et al. (2007) reported a similar response. Fruit characteristics (fruit mass, maturity and oil content) were not affected by the intra-row spacing at any harvest, consistent with previous reports in olive (Larbi et al., 2012; Trentacoste et al., 2015c; Díez et al., 2016). Thus, other aspects of orchard design such as inter-row spacing (Trentacoste et al., 2015a) or row-orientation (Trentacoste et al., 2015b) are more determinants of incident irradiance on canopy and consequently on fruit characteristics than intra-row spacing. It has been widely demonstrated in olive that fruit mass and oil content increase with increasing irradiance (Connor et al., 2009; Caruso et al., 2017).

Intra-row spacing is the primary determinant of the time required to form a continuous hedgerow. The objective of super-high density orchards is to increase production during the early years after establishment. In density trials, generally, greater production per ha has been obtained during the early years with high densities in apple (Robinson, 2003), orange (Wheaton et al., 1995), and olive (Pastor et al., 2007; Larbi et al., 2012; Díez et al., 2016). In our experiment the mean oil production ha^−1^ during the first 4 harvests (3–6th year) was linearly related with intra-row distance. During these 4 years accumulated oil production increased by 105 kg oil ha^−1^ for each 0.1 m reduction in intra-row distance (Figure 6). In orchard terms, accumulated oil production increased by 1.14 kg ha^−1^ for each additional tree per ha. But the early advantage of increased density, that increases productivity with a greater effective hedgerow length per ha, is gradually lost with time after planting.

The major impact of more closely spaced trees was to increase canopy leaf area ha^−1^ during the early years that increased radiation interception, and through it production. The yield advantage was, however, reduced after the 8th year because the more widely spaced trees (i) then nearly filled their allotted space and (ii) their more rounded-shape improved radiation penetration into the canopy. Díez et al. (2016) arrived at a similar conclusion over 14 year study period that revealed how hedgerow length and oil production were mainly affected by inter-row spacing, without influence of intra-row spacing.

## Conclusions

Optimal design of hedgerow olive orchards requires consideration of row orientation and spacing, intra-row density, soil and climatic conditions, water availability, training system, and cultivar reproductive and vegetative behavior. These features determine the time required to fill the hedgerow space and consequently early and later production. Early production is directly determined by radiation interception and distribution within the canopy that can be evaluated as hedgerow porosity or leaf area ha^−1^. Intra-row plant density is the major structural determinant of hedgerow closure and hence of early production.

In this experiment, tree growth and yield were not affected by intra-row spacing in the first 4 years after planting revealing absence of inter-tree competition. Subsequently, tree growth and yield were progressively reduced at decreasing intra-row spacing as tree volume and hedgerow porosity were reduced. Hedgerow closure had not been achieved after nine growing seasons at the widest tree spacing (2.5 m) compared with closure in 4–5 seasons at narrower tree spacing (1.0–1.63 m). In this way, orchards at narrow tree spacing achieved maximum oil production ha^−1^ 2 years earlier than those at wider spacing, with a consequent greater accumulated production of oil over study period (accumulated oil production increased from 9 to 12 t oil ha^−1^ reducing from 2.5 to 1.0 m the intra-row spacing). After the 4th harvest, however, oil production ha^−1^ was not affected by intra-row distance.

From an economic perspective, positive outcomes of oil production from narrow intra-row spacing in the early years must be weighed against greater cost of plants, planting and training during the first years, cash flow, and the currently uncertain longevity of these orchards in order to establish the most profitable tree density.

## Author contributions

MG and DC conceived and designed the experiment. MG and ET performed the evaluation. MG, DC, and ET analyzed the data and wrote the paper.

### Conflict of interest statement

The authors declare that the research was conducted in the absence of any commercial or financial relationships that could be construed as a potential conflict of interest.
